# Pancancer Analysis of Revealed *TDO2* as a Biomarker of Prognosis and Immunotherapy

**DOI:** 10.1155/2022/5447017

**Published:** 2022-09-09

**Authors:** Jing Cui, Yongjie Tian, Tianhang Liu, Xueyan Lin, Lanyu Li, Zhonghui Li, Liang Shen

**Affiliations:** ^1^Department of Oral and Maxillofacial Surgery, Jinan Stamotological Hospital, Jinan, 101 Jinglv Road, Shandong 250001, China; ^2^Central Laboratory of Jinan Stamotological Hospital, Jinan Key Laboratory of Oral Tissue Regeneration, Jinan, 101 Jinglv Road, Shandong 250001, China; ^3^Department of Gynecology, Shandong Provincial Hospital Affiliated to Shandong First Medical University, 324 Jingwu Weiqi Road, Jinan, Shandong 250021, China; ^4^Department of Gynecology, Central Hospital Affiliated to Shandong First Medical University, 105.jiefang road, Jinan, Shandong 250013, China; ^5^Department of Gynecology, Meihekou City Central Hospital, 2688 Kangmei Avenue, Meihekou city, Jilin 135000, China

## Abstract

**Background:**

Tryptophan 2,3-dioxygenase (TDO) encoded by *TDO2*, a rate-limiting enzyme in the kynurenine pathway, catabolizes tryptophan to kynurenine, evades immune surveillance, and promotes tumor growth. Although accumulating evidence suggests a crucial role of *TDO2* during tumor formation and development, systematic evaluation of *TDO2* across human cancers has rarely been reported.

**Methods:**

To shed more light on the role of *TDO2* in human cancer, we explored the expression profiles of *TDO2* and identified its prognostic value in pancancer analysis through TCGA, CCLE, and GTEx databases. We further utilized TCGA data to evaluate the association between *TDO2* and tumor immunological features, such as mismatch repair (MMR), tumor immune infiltration, immune checkpoint-related genes, tumor mutational burden (TMB), microsatellite instability (MSI), and DNA methyltransferase (DNMT).

**Results:**

*TDO2* exhibited different expression levels in various cancer cell lines. Frequently, *TDO2* was detected to be highly expressed in the majority of cancers. In addition, high *TDO2* expression was correlated with an unfavorable prognosis for patients in KIRP, LGG, TGCT, and UVM. Moreover, high *TDO2* expression level positively correlated with higher immune infiltration, especially dendritic cells. Additionally, there is a close relationship between *TDO2* and immune checkpoint-related gene markers, such as *LAIR1*, *CD276*, *NRP1*, *CD80*, and *CD86*. Finally, correlation analysis has demonstrated a high-correlation between *TDO2* and TMB, MSI, MMR, and DNMT of multiple cancer types.

**Conclusion:**

Therefore, our results suggest that *TDO2* can function as a potential prognostic biomarker due to its role in tumor immunity regulation.

## 1. Introduction

Globally, cancer remains an enormous health threat and the second most lethal cause of death [1]. Recently, immunotherapy, especially immune checkpoint inhibitor, has been becoming a hot research topic in field of cancer treatment [2]. With the rapid development of public databases, such as The Cancer Genome Atlas (TCGA) and The Genotype-Tissue Expression (GTEx), it is possible to explore novel immunotherapeutic target genes by searching for the relationship between expression and prognosis as well as various biological processes in pancancer [3, 4].

Tryptophan 2,3-dioxygenase (TDO) is encoded by the *TDO2* gene and functions as an initial, rate-limiting enzyme in the catabolism of tryptophan (Trp) via the kynurenine (Kyn) pathway and plays an essential role in the balance of systemic Trp levels [5]. Kyn, the major metabolism of Trp degradation, could activate aryl hydrocarbon receptor (AhR), inhibit antitumor immune, and accelerate the survival of cancer cells [6]. *TDO2* is found predominantly in the liver under physiological conditions [7]. Recently, increasing evidence has confirmed that *TDO2* is also involved in the occurrence and development of many cancers, such as colorectal, breast, esophagus, and bladder cancer [8–10]. Studies have found that liver metastasis of colon cancer could be accelerated by activating the *TDO2*-Kyn-AhR pathway [11]. However, most research on *TDO2* in cancer is limited to a given cancer type. To date, there are rare reports regarding a systematic pancancer analysis of *TDO2*.

Pancancer analysis aims to examine the commonalities and differences among the genomic and cellular alterations found across different tumor types and can help us explore the mechanisms and predict treatment outcomes from one tumor type to another tumor type. In this study, we utilized a variety of databases, including TCGA, CCLE, and GTEx to explore *TDO2* expression levels and their survival on pancancer data. Subsequently, we employed coexpression analysis of *TDO2* with immune cells infiltration, immune checkpoint-related genes *MMR*, *DNMT*, *TMB*, and *MSI* to elucidate the biological functions of *TDO2* across 33 types of cancers.

## 2. Materials and Methods

### 2.1. Data Collection and Progression

GTEx program provided expression data for 31 normal tissues, which could be downloaded through the GTEx portal. Based on the CCLE database, data were obtained for *TDO2* expression in 21 cancer cell lines. Using the GTEx and TCGA data, we examined the differences between *TDO2* expression levels in normal tissues and cancer. The level 3 RNA sequencing data and corresponding follow-up information were collected from the TCGA database. The values were performed to remove duplicates, then transformed using log2(TPM + 1) using the robust multichip average (RMA) method [12].

### 2.2. Cox Regression and Prognosis Analysis

Cox regression analysis was adopted to explore correlations between *TOD2* and major clinical outcome endpoints, such as overall survival (OS), disease-specific survival (DSS), and disease-free interval (DFI). Using the Kaplan–Meier method with R package survival, the survival curves were constructed for patients of each cancer type after classifying them into groups based on their *TDO2* expression in the best way. The time-dependent receive operating characteristic (ROC) curves were determined with the R packages survival ROC and survival [13] . A *P* value of less than 0.05 indicated significance.

### 2.3. Correlation of *TDO2* expression with Tumor Immune Microenvironment

The Tumor Immune Estimation Resource (TIMER) is a web-based, free database designed for comprehensive analysis of immune infiltrates in various types of cancer. It identifies immune cell types found in malignancies, such as dendritic cells, neutrophils, CD8+T cells, CD4+T cells, macrophages, and B cells. TIMER has already calculated immune cell infiltration scores from the TCGA data and published the results online. A correlation analysis was conducted between the infiltration data and the expression of *TDO2* here. Subsequently, a Spearman's correlation heat map analysis was performed to determine the association between immune checkpoint-related genes and *TDO2* gene expression in multiple cancers. TMB refers to the sum of all DNA mutations in tumor cells [14, 15]. The phenomenon of MSI is characterized by the addition or deletion of nucleotides in repeating DNA fragments [16]. Spearman's correlation analysis was conducted to evaluate the strength of correlation between *TDO2* expression and TMB or MSI. In addition, MMR can reduce chromosomal rearrangements, thereby preventing tumor genesis [17]. MutS homolog 6 (MSH6), MutS homolog 2 (MSH2), MutL homolog 1 (MLH1), epithelial cell adhesion molecule (EPCAM), and postmeiotic segregation increased 2 (PMS2) are five critical MMR genes [18]. The correlation of *TDO2* with MMR and DNMTs (DNMT1, DNMT2, DNMT3A, and DNMT3B) was investigated.

### 2.4. Statistics

Spearman's correlation tests were utilized by using R function correlation to determine the association between *TDO2* and a variety of immune-related targets, including immune cell infiltration, immune checkpoint-related genes, TMB, MSI, MMR, and DNMTs. Student's *t*-test was performed to determine differences in the *TDO2* expression levels between tumors and normal tissues using *t*-test function in R package. Graphs were generated by the R package ggplot2 and forest plot [19]. A *P* value of less than 0.05 indicated significance.

## 3. Results

### 3.1. Differential Expression of *TDO2* in Normal Tissues and Cancer

**Figure 1:**
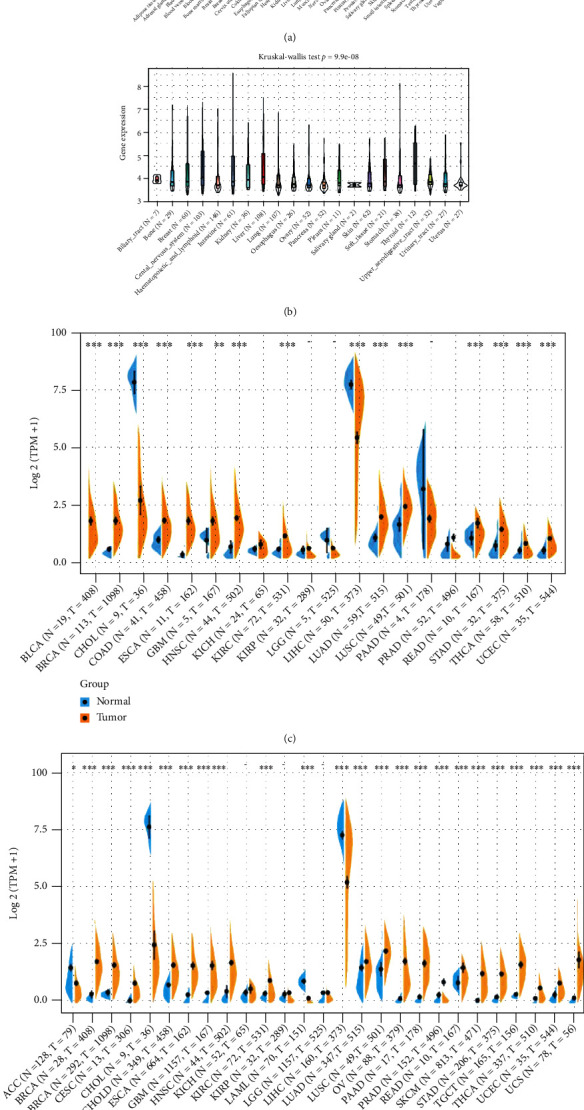
*TDO2* expression levels in different normal tissues and tumors. (a) *TDO2* expression in 31 normal tissues from GTEx database. (b) *TDO2* expression in 21 cancer cell lines from CCLE database. (c) different expression of *TDO2* between tumor and peritumor samples from TCGA database. (d) Different expression of *TDO2* between normal and tumor samples from TCGA and GTEx databases. Statistical analyses were performed using Kruskal–Wallis test. ^∗^*P* < 0.05, ^∗∗^*P* < 0.01, ^∗∗∗^*P* < 0.001.

Based on data from the GTEx, *TDO2* expression was deficient across all normal tissues, with the apparent exception of the liver and pituitary (Figure 1(a)). The *TDO2* expression level was elevated in various cancer cell lines (Figure 1(b)). Based only on TCGA data, the difference in expression level was statistically significant in 15 of 20 cancer types (KICH, KIRP, LGG, PAAD, and PRAD were five exceptions) (Figure 1(c)). Because the TCGA database contains a small number of normal specimens, we combined it with normal data from GTEx to analyze *TDO2* expression differences. The result showed significant differences in *TDO2* expression across 24 cancers, with higher *TDO2* expression in 20 cancer types (BLCA, BRCA, CESC, COAD, ESCA, GBM, HNSC, KIRC, LUAD, LUSC, OV, PAAD, PRAD, READ, SKCM, STAD, TGCT, THCA, UCEC, and UCS) and with lower *TDO2* expression in four cancer types (ACC, CHOL, LAML, and LIHC) as compared with the normal tissues (Figure 1(d)).

### 3.2. Prognosis Values Analysis of *TDO2*

**Figure 2:**
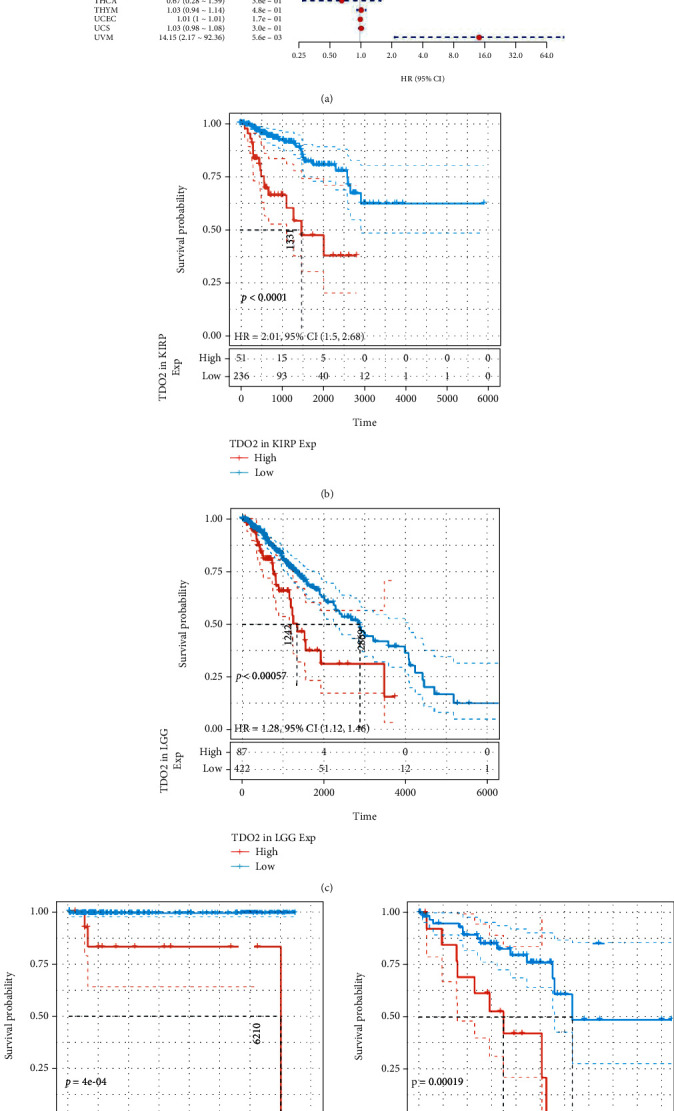
The correlation between *TDO2* expression and OS in pancancer. (a) Forest plot of OS associations in different cancer types of TCGA. (b) Kaplan–Meier analysis of the association between *TDO2* expression and OS in KIRP. (c) Kaplan–Meier analysis of the association between *TDO2* expression and OS in LGG. (d) Kaplan–Meier analysis of the association between *TDO2* expression and OS in TGCT. (e) Kaplan–Meier analysis of the association between *TDO2* expression and OS in UVM. *P* < 0.05 was considered significant, dash lines for 95% CI.

**Figure 3:**
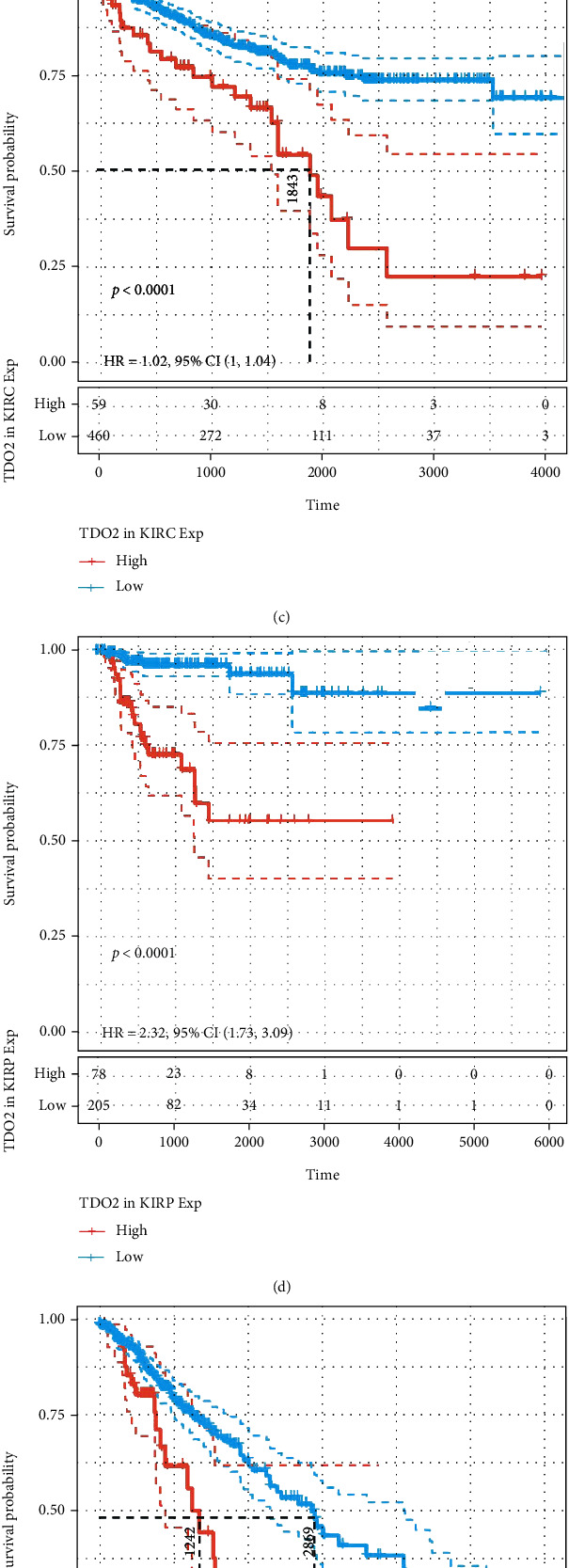
The correlation between *TDO2* expression and DSS in pancancer. (a) Forest plot of DSS associations in different cancer types of TCGA. (b) Kaplan–Meier analysis of the association between *TDO2* expression and DSS in KICH. (c) Kaplan–Meier analysis of the association between *TDO2* expression and DSS in KIRC. (d) Kaplan–Meier analysis of the association between *TDO2* expression and DSS in KIRP. (e) Kaplan–Meier analysis of the association between *TDO2* expression and DSS in LGG. (f) Kaplan–Meier analysis of the association between *TDO2* expression and DSS in UVM. *P* < 0.05 was considered significant, dash lines for 95% CI.

**Figure 4:**
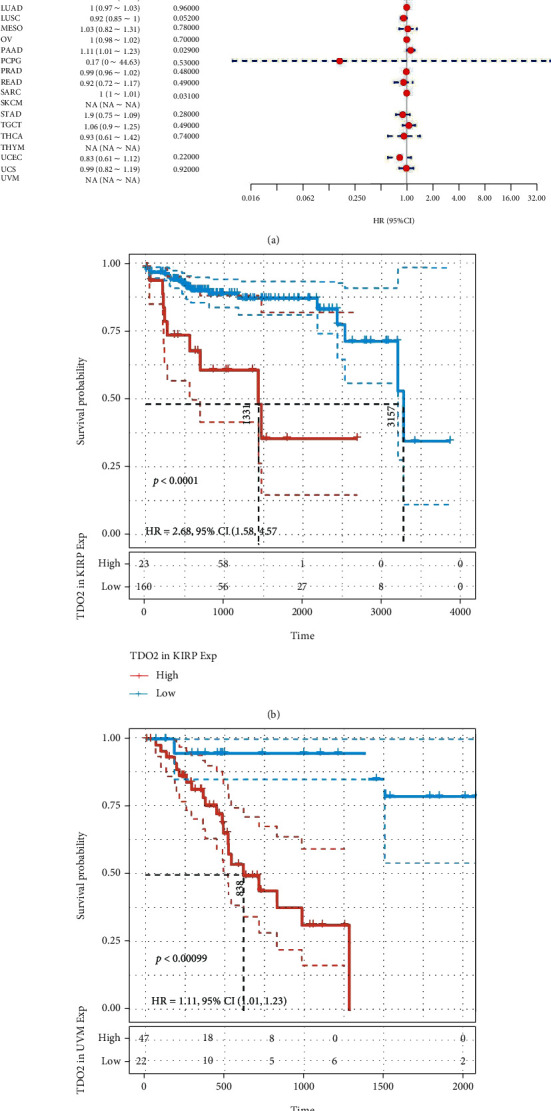
The correlation between *TDO2* expression and DFI in pancancer. (a) Forest plot of DFI associations in different cancer types of TCGA. (b) Kaplan–Meier analysis of the association between *TDO2* expression and DFI in KIRP. (c) Kaplan–Meier analysis of the association between *TDO2* expression and DFI in PAAD. *P* < 0.05 was considered significant, dash lines for 95% CI.

We first analyzed the TCGA data to evaluate correlations between *TDO2* expression levels and overall survival using univariate Cox regression. The HRs for *TDO2* achieved significance in KICH, KIRP, LGG, READ, UVM, and TGCT, among which the highest risk effect was observed in UVM (Figure 2(a)). When Kaplan–Meier analysis was performed on these cancer types, the differences in OS were statistically significant and patients with high *TDO2* expression had a poor outcome in KIRP, LGG, TGCT, and UVM (Figures 2(b)–2(e)). Considering nononcological mortality throughout the follow-up, we subsequently examined the associations between *TDO2* and DSS in 33 cancer types. There was a significant HR only in READ, LGG, KIRP, KIRC, KICH, and UVM (Figure 3(a)). According to the survival analyses of the six cancer types, patients with lower *TDO2* expression have a significantly better prognosis (Figures 3(b)–3(f)). Furthermore, we investigated their relationship and DFI across 33 cancer types. HR was found to be significant in the KIRP, PAAD, and SARC (Figure 4(a)). The survival curve showed that tumors recurred or metastasized sooner in KIRP and PAAD patients with high *TDO2* expression (Figures 4(b)–4(c)).

### 3.3. *TDO2* Expression and Immune Cell Infiltration Analyses

**Figure 5:**
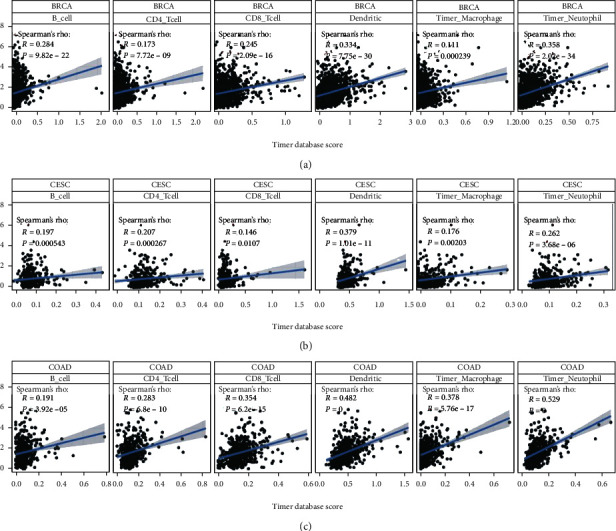
Correlation between *TDO2* expression and tumor infiltrations. Correlation between six immune cell infiltration scores (B cell, CD4+ T cell, CD8+ T cell, neutrophil, macrophage, dendritic cell) and *TDO2* mRNA expression in BRCA (a), CESC (b), and COAD (c). *P* < 0.05 was considered significant.

Our results suggest that *TDO2* could serve as a prognostic biomarker for several cancers. In the immune microenvironment, immune cells play essential roles and may affect tumor prognosis through tumor immunity [20]. This warrants further study to investigate the relationship between immune infiltration levels and *TDO2* expression. Our results show that *TDO2* expression correlated significantly with tumor purity in multiple cancer types. The BRCA, CESC, and COAD cancer were the top-ranking cancers. Dendritic cells were the most significant of six cell types in those three cancers (Figure 5).

### 3.4. Correlation of *TDO2* with TMB, MSI, and Immune Checkpoint-Related Genes MMR and DNMT

**Figure 6:**
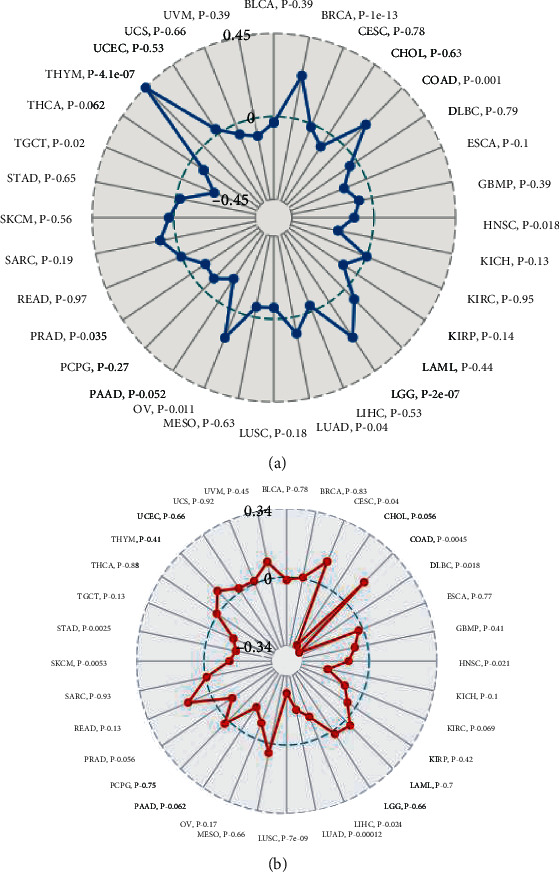
Radar map plotting the correlation of tumor mutation burden (TMB) (a) and microsatellite instability (MSI) (b) with *TDO2* expression across 33 cancer types.

**Figure 7:**
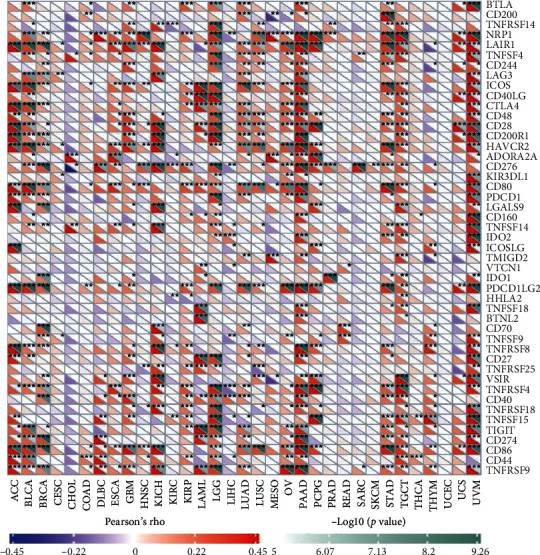
Correlation between *TDO2* expression levels and immune checkpoint related expression in multiple tumors. ^∗^*P* < 0.05, ^∗∗^*P* < 0.01, ^∗∗∗^*P* < 0.001.

**Figure 8:**
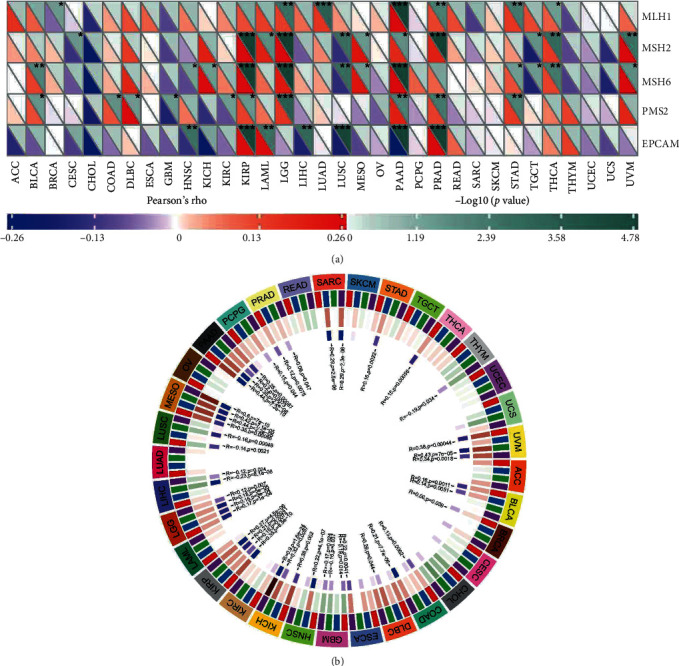
Correlation between MMR defects, methylation levels and *TDO2* mRNA expression level in various tumors from TCGA database. (a) Correlation between *TDO2* mRNA expression and mutation levels of five significant MMR genes. (b) Correlation between *TDO2* and four methyltransferases (DNMT1: red, DNMT2: blue, DNMT3A: green, DNMT3B: purple) mRNA levels. ^∗^*P* < 0.05, ^∗∗^*P* < 0.01, ^∗∗∗^*P* < 0.001.

TMB and MSI function as essential regulators on the occurrence and progression of tumors [21]. There was a significant relationship between *TDO2* and TMB in 10 of the 32 cancer types (BRCA, COAD, HNSC, LGG, LUAD, OV, TGCT, and TYUM). TYUM obtained the highest correlation coefficient, while TGCT obtained the lowest (Figure 6(a)). Furthermore, there was a significant relationship between *TDO2* and MSI in 9 out of 32 cancer types (CESC, COAD, DLBC, HNSC, KIRP, LIHC, LUAD, LUSC, SKCM, and STAD). The highest coefficients were obtained for COAD and the lowest coefficient was obtained for DLBC (Figure 6(b)). Further studies were carried out to determine the connection between *TDO2* and 47 immune checkpoint genes (Figure 7). *TDO2* expression was highly correlated with 37 genes in UVM, 36 genes in PAAD, 33 genes in LGG, and 32 genes in TGCT. Moreover, *TDO2* expression was associated with some specific immune checkpoint genes, including *LAIR1*, *CD276*, *NRP1*, *CD80*, and *CD86*. Mismatch repair (MMR), part of the DNA repair system, plays a crucial role in keeping genomes stable [22]. Our findings revealed that *TDO2* expression highly correlates with the MMR genes expression in different cancer types (KIRP, LGG, PAAD, and PRAD) (Figure 8(a)). Several recent studies have demonstrated that DNA methylation plays an essential regulatory function in tumorigenesis [23]. As shown in Figure 8(b), we identified the relationship between *TDO2* and four DNMTs. Many tumors express *TDO2* associated with four DNMTs, particularly PAAD, MESO, LGG, KRIP, KICH, GMB, and UVM. Mutation and DNA methylation in tumor cells may play a role in *TDO2*'s involvement in tumor development.

## 4. Discussion

In the present study, we identified that *TDO2* is highly expressed in 20 types of cancer, including BLCA, BRCA, CESC, COAD, ESCA, GBM, HNSC, KIRC, LUAD, LUSC, OV, PAAD, PRAD, READ, SKCM, STAD, TGCT, THCA, UCEC, and UCS, which are in line with previous findings [8–10, 24–28]. However, Wu et al. found that *TDO2* was overexpressed in HCC, and their overexpression was correlated with tumor progression and poor prognosis [29, 30], which contradicts our current results. On the other hand, Yu et al. investigated the expression of *TDO2* in HCC tissues compared with paired adjacent normal tissues and found that there was downregulation of *TDO2* expression in HCC, which agrees with our results [31]. This discrepancy may be due to the complex mechanisms of *TDO2* in HCC distinct from other tumors because under normal conditions, *TDO2* is predominantly highly expressed in the liver where it is the major metabolic location of Trp.

We found that high *TDO2* expression functions as a poor prognostic factor in multiple cancer types, such as KIRP, LGG, TGCT, and UVM. The previous study has proven that *TDO2* expression was highly elevated in colorectal cancer, and knockdown of *TDO2* significantly inhibited the proliferation, migration, and invasion of colorectal cancer cells [32]. In addition, *TDO2* was shown to overexpress in liver metastases from UVM and may be related to metastatic potential [33]. *TDO2* expression was upregulated in renal cell carcinoma and was associated with worse outcomes [34]. These results imply that aberrant *TDO2* expression plays a vital role in the development of cancer.

Our finding suggests that *TDO2* expression level is associated with the infiltration distribution of immune cells in various tumors. *TDO2* has been reported to suppress proliferation of T cells and induce T cell apoptosis, and in turn can alter the immune response [35]. Studies have proven that an overexpression of *TDO2* could activate AhR of immune cells and achieve immune escape [36]. It has been confirmed that *TDO2* is involved in mediating tumoral immune resistance, which raised considerable interest of targeting *TDO2* for cancer immunotherapy [37]. The treatment with a *TDO2* inhibitor could promote the function of dendritic cell and improve T cell mediated immune response, thereby diminishing tumor metastasis in mice [27]. The strong correlation between *TDO2* expression and some specific immune checkpoint gene expressions may be consequential to immune cell differentiation activated by AhR pathway.

TMB and MSI could serve as an emerging immunotherapy biomarker predictive of response to immune checkpoint inhibitors of tumors and guide personalized immunotherapy in the precision medicine era [38]. There are studies indicating that TMB has been proposed as an emerging, independent, and important predictive biomarker for cancer especially in non-small-cell lung carcinoma [39]. Multiple studies have found that MSI-H individuals have an improved overall prognosis and a favorable independent predictor. Our results showed that *TDO2* expression is associated with TMB in 10 different types of cancer and with MSI in 9 different types of cancer. This may suggest that *TDO2* expression level will influence the TMB and MSI of cancer, thereby impacting the patient's response to immune checkpoint inhibitors. This might supply some reference to explore the therapeutic effect of *TDO2* in immunotherapy.

Mutations and deficiency of MMR genes can result in genetic errors, contributing to tumorigenesis by causing genomic or microsatellite instability [40]. There is evidence that the MMR gene mutation is well positioned to be a predictor of tumorigenesis. Our results indicated that *TDO2* expression in human pancancer was closely associated with mutation rates of five MMR genes from pancancer analysis. The alteration of DNA methylation levels has been associated with tumorigenesis and immune evasion in cancer. According to our results, DNMTs and *TDO2* expression were specifically correlated in numerous types of cancer, indicating DNA methylation is likely to function in modulating *TDO2*.

To summarize, our pancancer analysis shows that *TDO2* expression was elevated in a variety of tumor types. Our findings demonstrate that *TDO2* could exert an oncogenic role and serve as a powerful cancer prognosticator of many cancers. Furthermore, we also found that expression of *TDO2* is correlated with immune cell infiltration, immune checkpoint-related genes, TMB, MSI, MMRs, and DNMTs. These findings will help us enhance the understanding of immune functions of *TDO2* in occurrence and development of various cancers and provide a new perspective on precise immunotherapy.

## Data Availability

All data are present in table and figures in this article; it can be available from the journal website.

## Abbreviations

AhR: Aryl hydrocarbon receptor
ACC: Adrenal cortical carcinoma
BLCA: Bladder urothelial carcinoma
BRCA: Breast invasive carcinoma
CCLE: Cancer Cell Line Encyclopedia
CHOL: Cholangiocarcinoma
COAD: Colon adenocarcinoma
DFI: Disease-free interval
DLBC: Lymphoid neoplasm diffuses large B-cell lymphoma
DSS: Disease-specific survival
DNMT: DNA methyltransferase
ESCA: Esophageal carcinoma
EPCAM: Epithelial cell adhesion molecule
GBM: Glioblastoma multiforme
GTEx: The Genotype-Tissue Expression
KICH: Kidney chromophobe
KIRC: Kidney renal clear cell carcinoma
KIRP: Kidney renal papillary cell carcinoma
Kyn: Kynurenine
LAML: Acute myeloid leukemia
LGG: Brain lower grade glioma
LIHC: Liver hepatocellular carcinoma
LUAD: Lung adenocarcinoma
LUSC: Lung squamous cell carcinoma
MESO: Mesothelioma
MLH1: MutL homolog 1
MMR: Mismatch repair
MSI: Microsatellite instability
MSH2: MutS homolog 2
MSH6: MutS homolog 6
OS: Overall survival
OV: Ovarian serous cystadenocarcinoma
PAAD: Pancreatic adenocarcinoma
PCPG: Pheochromocytoma and paraganglioma
PD-1: Programmed cell death protein 1 s
PMS2: Postmeiotic segregation increased 2
PRAD: Prostate adenocarcinoma
READ: Rectum adenocarcinoma
SARC: Sarcoma
SKCM: Skin cutaneous melanoma
STAD: Stomach adenocarcinoma
TCGA: The Cancer Genome Atlas
TDO: Tryptophan 2,3-dioxygenase
TGCT: Testicular germ cell tumor
THYM: Thymoma
TIMER: Tumor IMmune Estimation Resource
TMB: Tumor mutation burden
Trp: Tryptophan
UCEC: Uterine corpus endometrial carcinoma
UVM: Uveal melanoma.
